# The impact of eating habits on mood disorders (A prospetive study to show the importance of food on preventing mental heath disorders )

**DOI:** 10.1192/j.eurpsy.2024.1398

**Published:** 2024-08-27

**Authors:** N. Baabouchi

**Affiliations:** psychiatry, arrazi hospital, rabat, Morocco

## Abstract

**Introduction:**

Adopting a traditional healthy eating pattern is strongly associated with a more stable, adaptive, and serene mood. In contrast, adopting a modern and industrialized diet is linked to a higher incidence of anxiety and depressive disorders.

To prevent mood disorders, a varied diet rich in colorful fruits and vegetables is recommended. Studies show that the consumption of vegetables, whole grains, and fruits can help prevent the risk of major depression and anxiety disorders by more than 35%. A well-rounded plate, rich in micronutrients (trace elements, vitamins, minerals), is essential for the proper functioning of our brain and its emotional areas.

Our brain requires significant amounts of iron, zinc, magnesium, and vitamins B, E, D, and K. Unfortunately, our modern diet often lacks sufficient intake of these essential micronutrients. A deficiency in iron or zinc is associated with a significantly higher risk of major depression, and a lack of magnesium is a potential source of anxiety disorders.

Choosing a diet rich in micronutrients (whole grains, cereals, fresh fruits, and vegetables) can address potential deficiencies and contribute to a more adaptive and balanced mood. Similarly, carefully selected dietary supplements can prove to be effective.

**Objectives:**

it shows the importance of alimentation and her role on Primary and secondary prevention in depressive disorders.

**Methods:**

This poster is a prospective study done on 100 random people via a multi choice quizz, to see the impact of their food on their mental health .

**Results:**

in the making

**Conclusions:**

Food should today be universally considered as a potential risk factor or protective factor in depressive disorders. Since the recent decades, nutritional psychiatry has developed a field of research promising The International Society For Nutritional Psychiatry Research (ISNPR) who is a collective of doctors and researchers with the common objective of advance research and communication of nutritional medicine in the field of psychiatry. Cross-sectional epidemiological studies finding an association between diet quality and mental health in longitudinal studies, a step has been taken. The observational data have been widely replicated and documented in several meta-analyses and are supported by prospective studies studying the effectiveness of improving nutritional quality in the treatment of depression. It now appears necessary that in the near future psychiatrists must receive training on the impact of diet in psychiatric disorders including depression, and get into the habit of taking an interest in the eating habits of their patients, as well as their microbiota .

**Disclosure of Interest:**

None Declared

